# Revealing Patterns of Cognitive Function Trajectories in the Last Five Years of Life of Medicare Beneficiary

**DOI:** 10.1002/alz.090918

**Published:** 2025-01-09

**Authors:** Haiqun Lin, Weiyi Xia, Anum Zafar, Olga F Jarrín Montaner

**Affiliations:** ^1^ Rutgers, The State University of New Jersey, Newark, NJ USA; ^2^ Rutgers, The State University of New Jersey, Piscataway, NJ USA; ^3^ Rutgers, The State University of New Jersey, New Brunswick, NJ USA

## Abstract

**Background:**

Clinicians have observed various patterns of decline in cognitive function at the end of life. In a period of dynamic evolution in dementia care technology and delivery, tracking trends in cognitive function during last years of life provides a perspective on the need of patients and their caregivers and on demands on healthcare system. This work aims to characterize the functional decline in cognition during the last five years of life.

**Method:**

The analytic cohort included a 10% random sample consisting of 195,240 Medicare beneficiaries who died in 2019, aged fifty or older and continuously enrolled in Medicare during their last five years of life. The group‐based trajectory modeling approach for ordered categorical outcomes was adopted to characterize beneficiaries’ changes in cognitive function over the last five years: no impairment, memory loss, mild cognitive impairment, and dementia. Cognitive function status in each quarter of the last five years of life was assembled from linked administrative, claims, and assessment data.

**Result:**

Ten trajectory groups of cognitive function were identified and had a better value of Bayesian Information Criterion than either the 9‐class or the 11‐class solution. The 10 trajectory groups were categorized into four clusters: cognitively intact, mild cognitive impairment (MCI), steady decline to dementia, and early‐decline dementia. The intact cluster contains one trajectory group of 25% of beneficiaries who maintained their cognitive function throughout the last five years. The MCI cluster contains four trajectory groups with 37% in total of the beneficiaries who experienced slow decline in cognitive function and died with only MCI. The steady decline cluster contains four trajectory groups with 27% in total of the beneficiaries experienced steady decline in cognitive function and died with dementia. The early‐decline dementia cluster also contains only one trajectory group of 11% of the beneficiaries whose cognitive decline started more than five years before death. Sociodemographic and clinical factors associated with each cluster were assessed.

**Conclusion:**

This study identified distinct patterns of cognitive trajectories that varied in the timing and rate of cognitive decline during the last five years of life.

